# Phenotypic and genomic characterization of *Roseomonas mucosa*, an opportunistic pathogen with discrepancies among antimicrobial susceptibility testing methods

**DOI:** 10.1128/aac.01041-25

**Published:** 2026-01-21

**Authors:** Camille Cotet, Sebastien Galopin, Anne-Emeline Creach, Marwan Tenouri, Charlotte Le Pont, Nathalie Laquay, Jean-Christophe Giard, Simon Le Hello, François Gravey

**Affiliations:** 1 Department of Infectious Agents, Service of Bacteriology, Normandie Univ, UNICAEN, CHU de Caen Normandie, Caen, France; 2 Université de Caen Normandie, Univ Rouen Normandie, INSERM, Normandie Univ, DYNAMICURE UMR 1311 27003, Caen, France; 3 Normandie Univ, UNICAEN, CHU de Caen Normandie, Chirurgical Reanimation and Intensive Care Unit, Caen, France; 4 Department of Infectious Agents, Service of Bacteriology, Université de Caen Normandie, Univ Rouen Normandie, INSERM, Normandie Univ, DYNAMICURE UMR 1311, CHU Caen Normandie 27003, Caen, France; Universita degli Studi di Roma "La Sapienza", Rome, Italy

**Keywords:** *Roseomonas mucosa*, antimicrobial susceptibility testing methods

## Abstract

*Roseomonas mucosa* is an opportunistic bacterium found in clinical and environmental samples that primarily affects immunocompromised patients. Treatment is challenging owing to the lack of standardized susceptibility testing methods, breakpoints, and variable antimicrobial resistance profiles published. This study evaluated different antimicrobial susceptibility testing approaches and searched for new insights into resistance mechanisms, especially against third-generation cephalosporins. Antimicrobial susceptibility profiles of a panel of 17 *R*. *mucosa* strains were analyzed using disk diffusion method (DDM), broth microdilution (BMD) method, and MIC gradient strips. Discrepancies between susceptibility methods were further explored using beta-lactamase inhibitors, whole-genome sequencing, and transcriptomic analyses. Antimicrobial susceptibility testing revealed high susceptibility to aminoglycosides, fluoroquinolones, and carbapenems, while resistance to many beta-lactams was detected. Significant discrepancies were observed between the DDM and BMD methods, particularly with respect to the use of third- and fourth-generation cephalosporins and aztreonam. Genomic analysis identified two putative class-A and one class-C beta-lactamases within all strains. Transcription of one class A beta-lactamase, controlled by an *lysR* regulator, was significantly induced by cephalosporins and explained the phenotype observed. This study provides the antimicrobial susceptibility profiles against a large panel of antibiotics from 17 *R*. *mucosa* strains. It also explained the deep discrepancies between phenotypic approaches regarding cephalosporins.

## INTRODUCTION

Described for the first time in 1993 (1), bacteria that belong to the *Roseomonas* genus are now recognized as emerging opportunistic pathogens. This gram-negative coccobacillus, which is aerobic, slow growing, and pink pigmented, has been isolated from both clinical and environmental samples (2, 3). The reservoirs include surfaces, water, soil, plants, and human skin.

*Roseomonas mucosa* is the most common species isolated from clinical samples, mainly among immunocompromised patients with comorbidities such as malignant tumors, diabetes, systemic lupus erythematosus, and acquired immunodeficiency syndrome (4). Clinical infections may vary and include bacteremia, respiratory and skin infections, and peritonitis (5, 6). Invasive infections in immunocompetent patients, including meningitis (7) or dental root canal infection (8), have also been described.

Antimicrobial strategies for the treatment of these infections can be challenging because studies have focused mainly on case reports (9). There is no standard laboratory method for drug susceptibility testing specific to this genera (EUCAST-CASFM –-*Comité de l’Antibiogramme de la Société Française de Microbiologie* 2024; CLSI). This bacterium is typically described as susceptible to quinolones, aminoglycosides, and carbapenems but is frequently resistant to beta-lactams (ampicillin, piperacillin, and cephalosporins) (10). Besides, antimicrobial susceptibility varies among species; *R. mucosa* has shown higher antibiotic resistance rates (2).

In April 2022, a 63-year-old woman was hospitalized in our teaching hospital for the excision of a meningioma of the right ventricular junction. One week later, an external ventricular bypass was introduced due to intracranial pressure. After 2 weeks, she developed a persistent fever despite antibiotic treatment. Cerebrospinal fluid (CSF) analysis identified *R. mucosa*, leading to the diagnosis of cerebral ventriculitis. Empirical antimicrobial therapy was initiated with the combination of meropenem with intrathecal injections of amikacin. First-line antibiotics were switched for cefepime, which was categorized as “susceptible” regarding the diameters and EUCAST-CA-SFM guidelines (https://www.sfm-microbiologie.org/). Ten days later, the electroencephalogram revealed diffuse brain damage attributed to toxic encephalopathy due to the cefepime iatrogenic effect, confirmed by CSF analysis, which revealed an overdose of the antibiotic. Following a collegiate decision, treatment was changed to cotrimoxazole and ciprofloxacin for 3 weeks, leading to recovery.

In this study, we investigated the antimicrobial susceptibility profiles of a cohort of 17 *R*. *mucosa* strains alongside an in-depth genomic analysis. We combined phenotypic testing, whole-genome sequencing, and transcriptomics to understand discrepancies between antimicrobial susceptibility methods, leading to the description of three beta-lactamases in the genomes of *R. mucosa* species.

## MATERIALS AND METHODS

### Strains and growth conditions

The 17 strains were isolated between 1997 and 2022 in the teaching hospital of Caen Normandy, France. They were distributed among 14 *R. mucosa* samples (including the strain of the clinical case, id: 2733), one *Roseomonas giliardii* sample, and two *Roseomonas* spp. Species were identified via MALDI-TOF Biotyper sirius Brucker (Billerica, MA, USA). Most of the strains were isolated from human samples (*n*=13), notably from blood cultures (*n*=5), meconium (*n*=5), cerebrospinal fluid (*n*=2), and finger swabs (*n*=1). The four environmental isolates were collected from surfaces or gloves by the hospital hygienist department (Table S1).

Optimal growth conditions were assessed by incubating cultures at 35°C for 24 and 48 h under aerobic and anaerobic conditions using Mueller–Hinton medium or Muller–Hinton fastidious agar.

### Antimicrobial susceptibility testing and determination of the minimum inhibitory concentrations

Twenty-eight antibiotics were tested via the disk diffusion method (DDM). Inhibition diameters were interpreted using EUCAST-CASFM 2024 breakpoints for *Acinetobacter* spp. Minimum inhibitory concentrations (MICs) of 24 molecules were determined via the broth microdilution (BMD) method with FRAM2GN and EUMDROXF Sensititre plates (Thermo Fisher Scientific, Waltham, MA, USA) or manually. For 16 molecules, the susceptibility profiles were determined via both DDM and BMD methods (Table S2). MICs of cefiderocol were determined using UMIC test kits (Bruker, Germany) according to the manufacturer’s instructions. Considering the absence of breakpoints for *Roseomonas* species, in both EUCAST and CA-SFM referential, MICs were interpreted according to breakpoints available in the “nonrelated species PK/PD breakpoints” section of the EUCAST-CASFM 2024 guidance document. In case of discrepancies between methods only, results obtained with BMD were considered.

MIC gradient strips were used to compare the MICs of cefotaxime and ceftriaxone with those obtained with BMD approaches. MIC gradient MICs from two manufacturers, Biomerieux (Marcy l’étoile, France) and Liofilchem (Roseto degli Abruzzi, Italy), were measured according to their recommendations.

Plates were incubated at 35°C for 48 h for all the antimicrobial susceptibility approaches.

The associations between MICs and diameters were visualized using R version 4.4.2 on RStudio software version 4.2.1 and ggplot2 version 3.5.1 (11).

### Genomic analyses

Genomes of the seventeen strains were sequenced using the NextSeq 500 platform, generating 150 pb paired-end reads (Illumina Technology, San Diego, CA, USA) after DNA extraction via the Magna Pure system (Roche, Bate, Suisse) and library preparation by the Nextera Kit according to the manufacturer’s instructions (Illumina, San Diego, CA, USA) at the “*plateforme de microbiologie mutualisée”* (p2m, Institut Pasteur, Paris, France). Quality control of the reads was performed with FastQC https://www.bioinformatics.babraham.ac.uk/projects/fastqc/ and MultiQC (12). *De novo* assembly was carried out via SPAdes v3.12 (13). The quality of the genomes was assessed using QUAST (14). Species determination was performed using the rMLST tool (15). Genome annotation was performed with Prokka (16). Antimicrobial resistance genes were looked for in both Resfinder (17) and CARD (18) databases; all questioned in December 2024.

Sixty-four additional genomes of *Roseomonas* spp. were downloaded from NCBI to realize an average nucleotide identity (ANI) approach between the genomes from this study and genomes available in RefSeq. Details are available in Table S3. ANI was performed using the mash dist algorithm (19); the distance matrix obtained was converted into a neighbor-joining tree using the Phylogeny Inference Package (20) and visualized using iTOL version 7 (21).

Sequences of the three beta-lactamases as well as the *lysR* regulator were *in silico* translated to assess the diversity of these proteins within the genomes collection.

### Exploration of the effects of beta-lactamase inhibitors on the MICs of third-generation cephalosporins

MICs of cefotaxime and ceftriaxone were assessed by adding clavulanic acid in the Mueller-Hinton (MH) medium using clavulanic acid/amoxicillin MIC gradient strips Biomerieux (Marcy l’étoile, France), or via a specific MH plus cloxacillin (250 mg/L) Biomerieux (Marcy l’étoile, France). MICs were measured using MIC gradient strips; plates were incubated aerobically at 35°C for 48 h.

### Transcriptomic response of *R. mucosa* to cefotaxime exposure

Strain 2733 was grown on MH plates with ceftriaxone and cefotaxime MIC gradient strips. After 48 h at 37°C, cells near the strips (with antibiotics) and those from the plate periphery (without antibiotics) were harvested. Total RNA was extracted using the Maxwell RSC miRNA Tissue Kit (Promega, Madison, WI, USA) and quantified via NanoDrop (Thermo Fisher Scientific, Waltham, MA, USA). cDNA synthesis (0.1 μg) and RT-qPCR were performed using the Platinum SYBR Green One-Step qRT-PCR Kit (Invitrogen, Waltham, MA, USA) according to the manufacturer’s instructions. The expression of beta-lactamase encoding genes (*ros1*, *ros2*, and *ecl_03254*) as well as the *lysR* was calculated by comparing transcription levels with and without antibiotics, using *gyrA* as an internal control. Experiments were repeated at least three times. Ratios were compared using the ΔΔCt method (22); statistical significance was assessed using Student’s *t*-test (*P* < 0.05). Sequences of the primers used are available in Table S4. *Ros2* gene, for which its expression was associated with ceftriaxone and cefotaxime exposure, was cloned with its promoter and expressed in *Escherichia coli* to further explore its contribution to the observed phenotype.

### Construction of multicopy plasmid containing ros2 beta-lactamase

The *ros2* gene from *R. mucosa* strain 2733, with or without its own promoters, was amplified by PCR using primers listed in Table S5. Each PCR product was then cloned into the low-copy number overexpression plasmid pBAD202 Directional TOPO (Invitrogen, Carlsbad, CA, USA) and transformed into *E. coli* Top10 recipient cells (Invitrogen) according to the manufacturer’s instructions. *E. coli* cells carrying pBAD202 recombinants containing correctly oriented fragments were selected on LB plates with 50 mg/L of kanamycin. Inhibition diameters of amoxicillin, amoxicillin + clavulanic acid, cefotaxime, and ceftriaxone were measured using *E. coli* cells carrying pBAD202 with and without ros-2 cloned with its promoter.

## RESULTS

### Species determination

Based on the mass spectrometry results, 14 strains belonged to the *R. mucosa* species, one to *R. gilardii* species (1793), and two (3994, 11153) were only identified at the genus level. Genomic analyses revealed that all the strains belonged to the *R. mucosa* species (Table S1). ANI approaches were in accordance with the rMLST taxonomic assignation, Fig. S1.

### Antimicrobial profile determination

The optimal growth condition was an aerobic atmosphere on MH agar and was used during all the following experimentations.

Considering the DDM results, several antibiotics were active including aminoglycosides: amikacin, gentamicin, and tobramycin (percentage of sensitivity [PS] 100%); fluoroquinolones: ciprofloxacin and levofloxacin (PS 100%), carbapenems imipenem and meropenem (PS 100%); ticarcillin-clavulanic acid (PS 100%), cefepime (PS:100%), and cotrimoxazole (SP 100%; Table S6). In contrast, all the strains were resistant to piperacillin-tazobactam and ceftazidime (Table S6). BMD confirmed the efficacy of aminoglycosides (MIC_50/90_ of gentamicin 0.125 mg/L, MIC_50/90_ of tobramycin ≤0.5 mg/L; and MIC_50/90_ of amikacin ≤2 mg/L) and cotrimoxazole (MIC_50/90_ ≤ 1 mg/L; Table 1; Table S7). Notably, MICs of colistin were high (MIC_50/90_ 16 mg/L), whereas the MICs of eravacycline were low (MIC_50/90_ 0.06 mg/L; Table S7). For both antibiotics, no clinical interpretations were made due to the absence of PK/PD breakpoints in the CA-SFM 2024 reference.

**TABLE 1:** Inhibitory diameters and MICs for the 17 strains of *R. mucosa* according to the CASFM-EUCAST criteria

Antibiotic	MIC (mg/L)			Diameter (mm)			
	MIC50	MIC90	% S^*a*^	Minimum	Maximum	Median	% S^*e*^
Piperacillin-tazobactam^*b*^	1,024	1,024	0	6	7	6	0
Temocillin^*c*^	>32	>32	0	6	7	6	NA^*f*^
Ceftriaxone^*b*^	4	16	40	30	40	36	NA
Cefotaxime^*b*^	8	32	20	18	30	25	NA
Cefepime^*c*^	16	16	10	15	25	19	100
Ceftazidime^*c*^	32	32	0	6	7	6	0
Ceftazidime-avibactam	>32	>32	0	NA	NA	NA	NA
Ceftolozane-tazobactam	>32	>32	0	NA	NA	NA	NA
Ertapenem^*c*^	0.125	0.25	100	36	40	38	NA
Imipenem^*c*^	≤1	≤1	100	37	40	39	100
Imipenem-relebactam	0.5	1	100	NA	NA	NA	NA
Meropenem^*a*^	0.5	0.5	100	28	40	40	100
Meropenem-vaborbactam	0.25	0.25	100	NA	NA	NA	NA
Aztreonam^*c*^	32	32	0	14	39	20	NA
Cefiderocol	16	16	0	NA	NA	NA	NA
Amikacin^*c*,*d*^	≤2	≤2	NA	22	40	40	100
Gentamicin^*b*^	0.125	0.125	100	35	40	40	100
Tobramycin^*c*^	≤0.5	≤0.5	100	34	40	40	100
Ciprofloxacin^*b*^	0.25	0.5	1	21	40	40	100
Tigecycline^*c*^	≤0.5	≤0.5	100	28	40	35	NA
Cotrimoxazole^*c*^	≤1	≤1	100	20	40	39	100

^*a*^ Susceptibility interpreted according to breakpoints proposed in the “nonrelated species PK/PD breakpoints” section of the referential EUCAST-CASFM 2024.

^*b*^ MICs determined by manual broth dilution methods using the reference strain *E. coli* ATCC29212.

^*c*^ MICs determined via Sensititre on the EUMDROXF, FRAM2GN plates (Thermo Fisher).

^*d*^ MICs were interpreted via the three EUCAST-CASFM 2024 breakpoints:* Acinetobacte*r spp.

^*e*^ Diameters of inhibition were interpreted via the three EUCAST-CASFM 2024 breakpoints:* Acinetobacte*r spp.

^*f*^ NA, not available.

Among the beta-lactams, the efficacies of new beta-lactam–beta-lactamase inhibitor combinations as well as new molecules were variable. All the strains were resistant to ceftazidime-avibactam (MIC_50/90_ > 32 mg/L) or ceftolozan-tazobactam (MIC_50/90_ > 32 mg/L; Table 1; Table S7). The combinations of imipenem-relabactam and meropenem-vaborbactam were effective (MIC_50/90_ of 1 mg/L and MIC_50/90_ of 0.25/1 mg/L, respectively; Table 1; Table S7). In contrast, temocillin, ceftazidime, piperacillin-tazobactam, and cefiderocol were ineffective (MIC_50/90_ > 32 mg/L, MIC_50/90_ > 32 mg/L, MIC_50/90_ 1,024 mg/L, and MIC_50/90_ 16mg/L, respectively; Table 1; Table S7).

**Fig 1:**
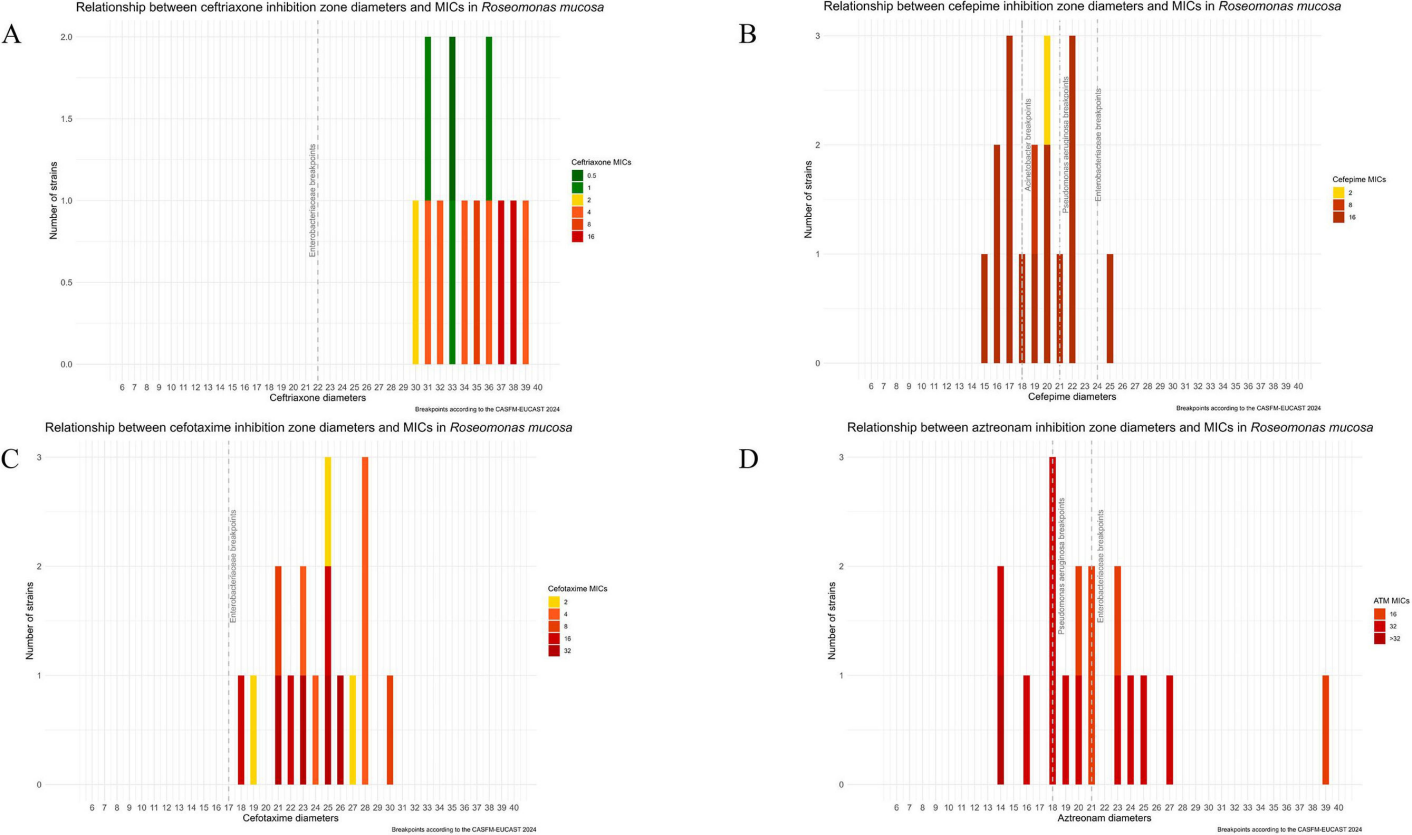
Distribution between DDM and BMD for the *R. mucosa* cohort according to the CASFM-EUCAST 2024 breakpoints. (**A**) Ceftriaxone. (**B**) Cefepime. (**C**) Cefotaxime. (**D**) Aztreonam.

Whereas all the strains were categorized as sensitive to cefepime according to the DDM, the susceptibility rate dropped to 10% regarding the BMD results (Table 1). Furthermore, the sensitivity rates of ceftriaxone and cefotaxime were 40% and 20%, respectively (Table 1; Table S8). These low susceptibility rates were unexpected considering the inhibition diameters observed for both molecules: 36 mm for ceftriaxone and 25 mm for cefotaxime (Fig. S2). The MICs of ceftriaxone and cefotaxime were confirmed via MIC gradient strips from two different manufacturers. For cefotaxime, the MIC_50/90_ were higher with the MIC gradient strips rather than with the BMD test (>32 mg/L; Table S8). For ceftriaxone, the MICs obtained with the MIC gradient were slightly lower than those obtained with BMD (MIC_50/90_ 2/4 mg/L with Biomerieux and MIC_50/90_ 4/8 mg/L with Liofilchem; Table S8). Notably, colonies that were grown at relatively high MICs were tested again by DDM and presented the same original diameter, suggesting that the strain adapted to elevated concentrations of cephalosporins rather than being resistant mutants. According to these results and for the 16 molecules tested with both DDM and BMD, the distribution of diameters and MICs was studied (Fig. S3). For ceftriaxone (Fig. 1A), cefepime (Fig. 1B), cefotaxime (Fig. 1C), and aztreonam (Fig. 1D), the diameter measured was not predictive of the MICs.

### Identification of two putative class A and one class C beta-lactamase

No gene encoding for a beta-lactamase was found in the genomes according to ResFinder and Card databases. More detailed explorations of the genome annotation of the 17 strains revealed the presence of three proteins that harbored a putative beta-lactamase motif (Table S9). The first gene, PRJNA1137846: AB3X21_00595, encoded for a serine hydrolase with beta-lactamase activity and will be hereafter named ROS-1. The corresponding gene was located between a metalloendopeptidase and a voltage-gated chloride channel-encoding gene. The best match in the card database of this protein was OKP-C-1 beta-lactamase, with an identity rate of 30% and coverage of 44%, followed by several variants of SHV beta-lactamase from *Klebsiella pneumoniae*, namely, SHV-156, SHV-157, and SHV-62, with identity rates between 30% and 31% and coverage between 49% and 50%. The second protein, PRJNA1137846: AB3X21_17530, was annotated as a class A beta-lactamase and will hereafter be named ROS-2. The corresponding gene was located between a gluconate 2-dehydrogenase subunit 3 family and a LysR family transcriptional regulator. This putative beta-lactamase shares approximately 50% identity with several class A beta-lactamases, including the PEN beta-lactamase recovered from *Burkholderia multivorans* strains, CARB from *Acinetobacter baumannii*, *Psychobacter maritimus*, or *Oligella urethralis*, and BOR-1 from *Bordetella bronchiseptica*. The protein sequence of the LysR regulator (PRJNA1137846: AB3X21_17535) shares more than 80% identity with the AmpR/LysR transcriptional activator identified in *Pseudoroseomonas coralli*, *Acetobacteraceae bacterium,* and *Aliidongia dinghuensis*. A class C beta-lactamase-encoding gene was also found. This gene was next close to the *ECL_03254* gene (between 99% and 100% identity and 100% coverage), described among *Enterobacter cloacae* complex strains.

To experimentally verify the beta-lactamase activity of the *rso2* gene product, we cloned this locus into a strain of *E. coli*. This heterologous complemented strain proved to be more susceptible to amoxicillin than the wild-type strain when clavulanic acid was added, showing that Ros2 belonged to a class A enzyme (Table S10).

The sequence of the LysR transcriptional factor was similar among all the strains. Two different sequences of the protein ECL_03254 were found, distant by only one amino acid substitution p.Ala85Thr (Table S9). Three sequences of ROS-2 were found with a maximum of three amino acid substitutions p.Ser42Gly, p.Val252Ala, and p.Gly282Ser (Table S9). On the opposite, ROS-1 was very variable with a total of 10 different sequences including six proteins with a deletion of the C-terminal extremity (Table S9). Considering the MICs measured and the integrity of the proteins, it seems that ROS-1 did not have an impact on the phenotype observed as the MICs were uniformly distributed regardless of the integrity of this protein (Table S9). Those three beta-lactamases were found in all the 41 genomes of *R. mucosa* (NCBI + this study; Table S11). The sequence of ROS-2 was conserved, and all the genes presented the same length with an identity rate from 99.2% to 100%. The sequence of ROS-1 was less conserved, with gene length between 887 and 1,266 nucleotides and identity percentages from 97.5% to 100%. Considering the ECL_03254_betalactamase, all the genes presented the same length with an identity rate from 99.5% to 100%.

### Exploration of discrepancies between susceptibility testing methods

Considering the presence of both a Class-C and two Class-A beta-lactamases, the efficiency of beta-lactamase inhibitors on the MICs was assessed. Cefotaxime MICs were measured on an MH agar associated with 250 mg/L cloxacillin to counteract the effect of a putative beta-lactamase with a cephalosporinase activity, but MICs did not change (data not shown). The combination of acid clavulanic/amoxicillin plus cefotaxime presented an MIC of 0.75 mg/L versus greater than 32 mg/L without clavulanic acid, which indicated a reduction of more than five dilutions of the MICs of cefotaxime alone (Fig. S4). The MICs of ceftriaxone decreased from 2 mg/L to 1 mg/L after the addition of clavulanic acid/amoxicillin. These results suggest the involvement of a type A-beta lactamase in the observed phenotype. This fact was also suspected based on the inhibition diameters of amoxicillin-clavulanic acid and ticarcillin-clavulanic acid, which were all larger than those of amoxicillin and ticarcillin alone (Table S6).

### Transcriptional level of beta-lactamases in the presence of cefotaxime or ceftriaxone

**Fig 2:**
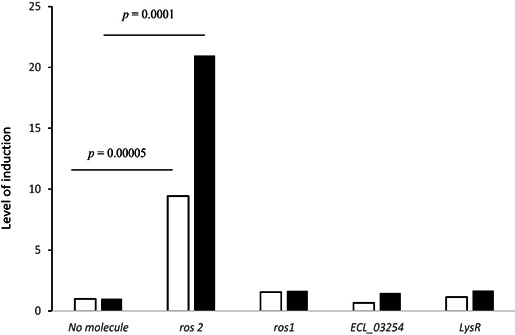
Levels of induction of *ros2*, *ros1*, *ECL_03254*, and *lysR* gene expression when cells were cultured in the presence of ceftriaxone (white bars) and cefotaxime (black bars; see Materials and Methods section) compared with the expression level in the absence of antibiotics (normalized to 1).

As shown in Fig. 2, the expression of the *ros2* gene increased 9- and 20-fold in the presence of the third-generation cephalosporins ceftriaxone and cefotaxime, respectively. At the same time, the transcription of the two other beta-lactamases (*ros1* and *ECL_03254* like) as well as the *lysR* genes was not affected by the presence of these molecules.

Altogether, (i) the fact that truncated ROS-1 had no effect on the MICs measured, (ii) the absence of cloxacillin effect on the MICs, and (iii) the transcriptomics results demonstrated that ROS-2 was responsible for the phenotype observed.

## DISCUSSION

Little is known about the appropriate antimicrobial therapy and which molecule should be tested in microbiology laboratories to respond to *R. mucosa* infections, whereas this opportunistic pathogen has been associated with various community- and hospital-acquired infections (23).

Our results mostly agree with those of Han et al., who analyzed 36 strains (and 2 ATCC) of *Roseomonas* species (2). Where all the strains were susceptible to amikacin and ciprofloxacin, most of them were also susceptible to ticarcillin-clavulanate (31/38), whereas susceptibility rates to cotrimoxazole were lower (29% [11/38]). Han et al. also warned against the poor efficacy of third-generation cephalosporins (ceftriaxone S = 18/38; ceftazidime S = 0/38). Moreover, the results from Nariyama and coworkers are consistent with our findings, except for the use of cotrimoxazole, which was inefficient toward the strain causing peritonitis infection (5). The overall susceptibility patterns of several previously reported cases are in agreement with our findings (4, 6, 24, 25). Nevertheless, none of the published studies address the discrepancies regarding cefotaxime and ceftriaxone DDM/BDM results despite their common use in antimicrobial therapy strategies.

Despite the absence of results within the resfinder (26) and card databases (18), *in silico* analyses revealed the presence of three putative beta-lactamases in the *R. mucosa* genomes. Among them, ROS-2 was responsible for the discrepancies between inhibition diameters and MICs for cefotaxime and ceftriaxone. Interestingly, a class D beta-lactamase was found among *Roseomonas fluvialis* genomes (27). This oxacillinase was not found in any genomes of *R. mucosa*, from this study either from NCBI.

Our study highlighted the necessity of considering MICs and not using the DDM to determine the antimicrobial susceptibility of this bacterium, especially for cephalosporins. The discrepancy between three susceptibility methods, namely, the DDM, BMD, and MIC gradient strips, is a crucial issue in clinical microbiology. DDM and MIC gradients are commonly used to assess the antimicrobial susceptibility profiles of bacteria. In this context, they yielded unreliable results, as demonstrated in this study, posing challenges for the treatment of infections. Discrepancies between DDM and the MIC gradient (both based on an antibiotic gradient in the agar plate) could be correlated with the concentration required for the new ROS-2 beta-lactamase induction. Moreover, the impact of the breakpoints used to interpret MICs is crucial, mainly for cefepime; with a MIC50/90 at 16mg/L, all strains were categorized as resistant considering PK/PD values, but could have been considered “sensible” with the *Acinetobacter* spp. breakpoints.

With the incorporation of *ros-2* into the pBAD plasmid, the *E. coli* TOP-10 strain had a phenotypic class A penicillinase phenotype. Unfortunately, no modification of the MICs of third-generation cephalosporins was observed. This observation could be explained by the fact that the transcriptional regulator *lys-R* was not included in the plasmid, whereas this protein is crucial for ros-2 hyper-expression, as has been demonstrated by the transcriptomic analyses.

### Conclusion

Significant discrepancies between susceptibility methods performed by DDM and those based on the determination of MICs were demonstrated for *R. mucosa*. As a consequence, in line with the official guidelines CA-SFM and EUCAST, MICs must be used instead of diffusion methods to interpret the susceptibility of strains to avoid treatment failure.

## Data Availability

Genomes are publicly available under BioProject accession number PRJNA1137846.

## SUPPLEMENTAL MATERIAL

Fig. S1 aac.01041-25-s0001.svg Phylogenetic approach.

Fig. S2 aac.01041-25-s0002.tif Inhibitory diameters and MICs of ceftriaxone and cefotaxime using MIC gradient strips of the 17390 *R. mucosa* strain.

Fig. S3 aac.01041-25-s0003.tif Distribution between DD and BMD for the *R. mucosa* cohort according to the CASFM-EUCAST 2024 breakpoints regarding 11 molecules.

Fig. S4 aac.01041-25-s0004.tif Inhibition diameters and MICs of cefotaxime using DDM and MIC gradient strips alone or combined with clavulanic acid on the 2733 clinical strain.

Supplemental material aac.01041-25-s0005.docx Supplemental figure legends.

Supplemental tables aac.01041-25-s0006.xlsx Tables S1 to S11.

ASM does not own the copyrights to Supplemental Material that may be linked to, or accessed through, an article. The authors have granted ASM a non-exclusive, world-wide license to publish the Supplemental Material files. Please contact the corresponding author directly for reuse.
