# Supplementary figures and images for: Phenotypic and genomic characterization of Roseomonas mucosa, an opportunistic pathogen with discrepancies among antimicrobial susceptibility testing methods
Source: Antimicrob Agents Chemother. 2026 Jan 21;70(3):e01041-25. doi: 10.1128/aac.01041-25 (PMC12959163; doi:10.1128/aac.01041-25)

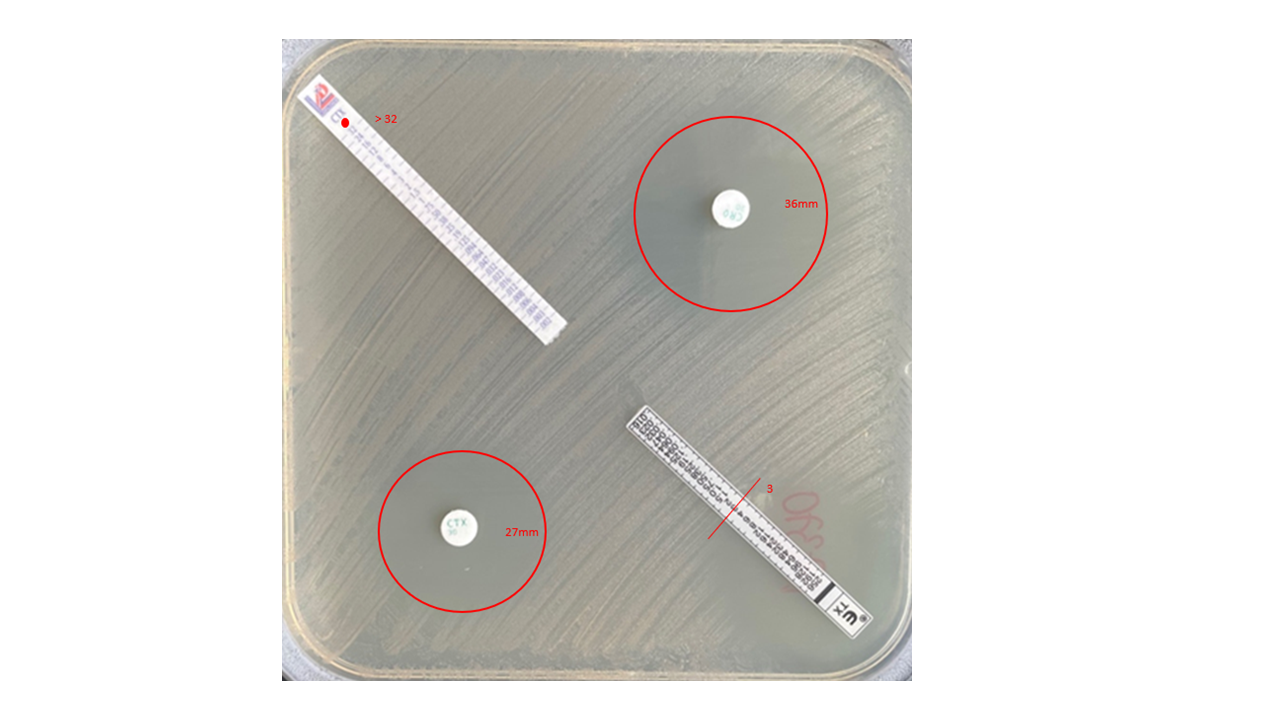

Supplement: Fig. S2 — Inhibitory diameters and MICs of ceftriaxone and cefotaxime using MIC gradient strips of the 17390 R. mucosa strain. [file aac.01041-25-s0002.tif]

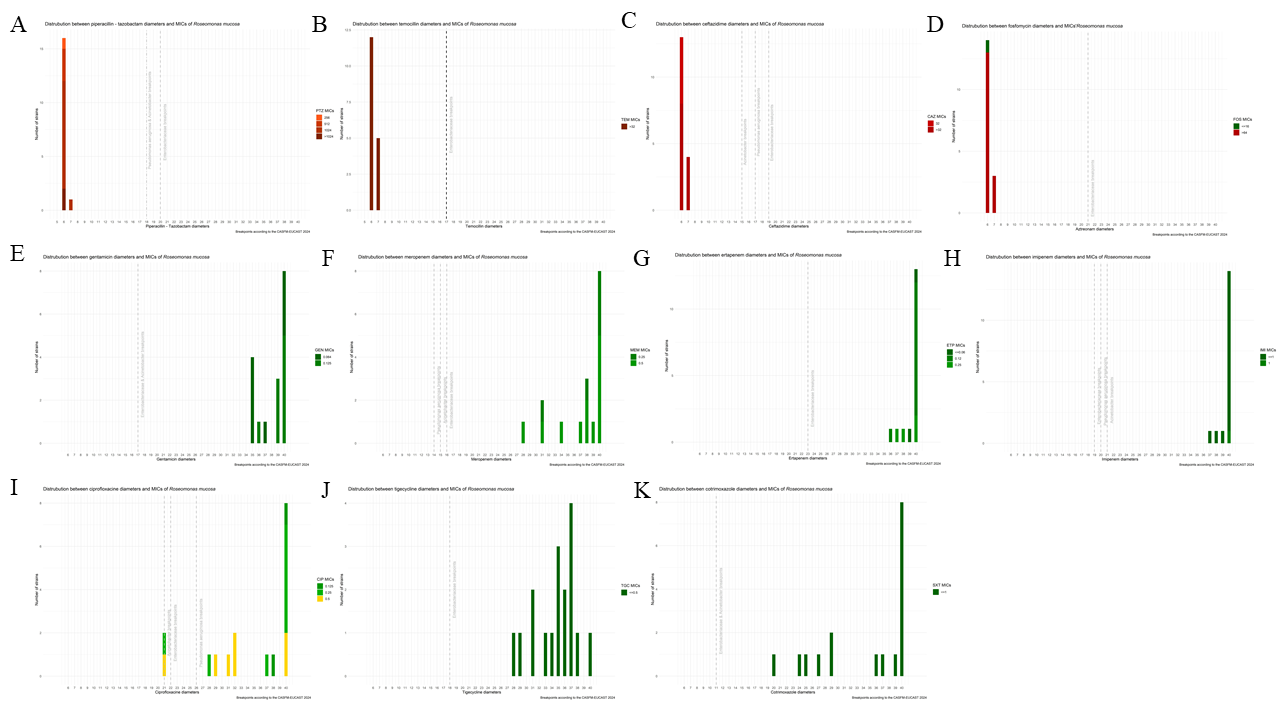

Supplement: Fig. S3 — Distribution between DD and BMD for the R. mucosa cohort according to the CASFM-EUCAST 2024 breakpoints regarding 11 molecules. [file aac.01041-25-s0003.tif]

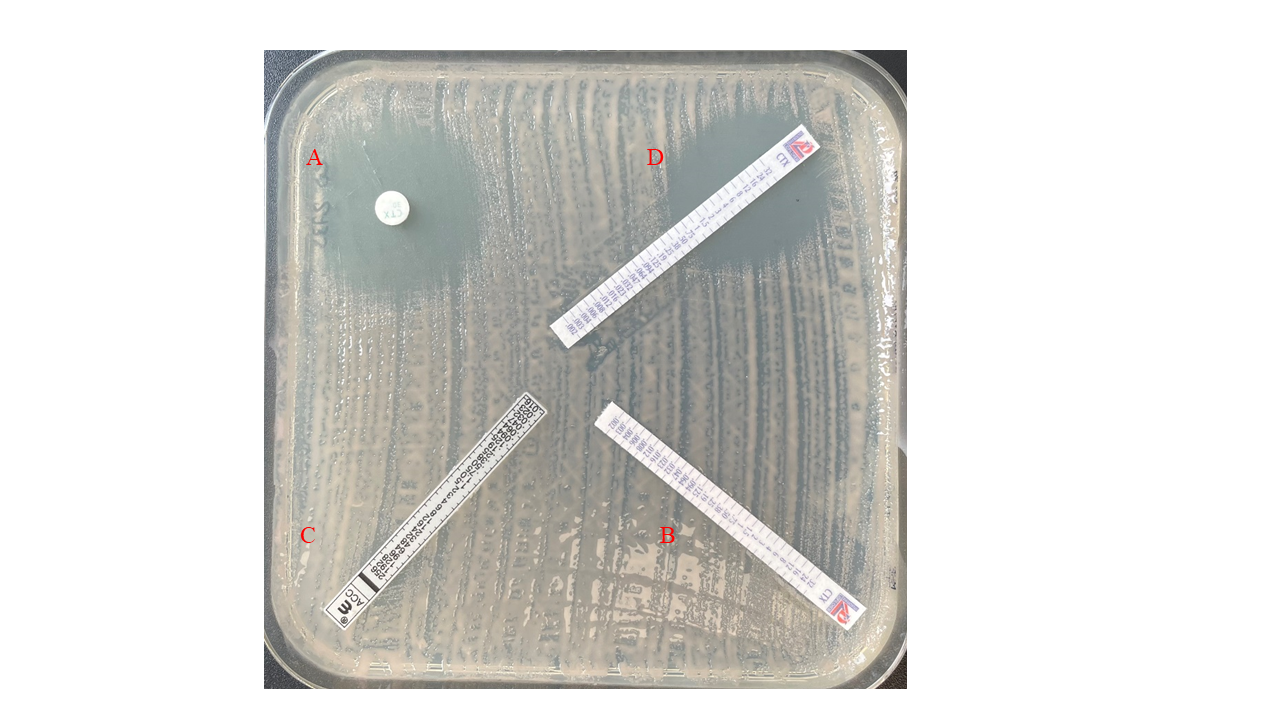

Supplement: Fig. S4 — Inhibition diameters and MICs of cefotaxime using DDM and MIC gradient strips alone or combined with clavulanic acid on the 2733 clinical strain. [file aac.01041-25-s0004.tif]
