# Supplementary material for: Phenotypic and genomic characterization of Roseomonas mucosa, an opportunistic pathogen with discrepancies among antimicrobial susceptibility testing methods
Source: Antimicrob Agents Chemother. 2026 Jan 21;70(3):e01041-25. doi: 10.1128/aac.01041-25 (PMC12959163; doi:10.1128/aac.01041-25)
Supplement: Supplemental material — Supplemental figure legends. [file aac.01041-25-s0005.docx]

Supplementary figures:

Figure S1: Neighbor joining tree of 81 *Roseomonas* spp genomes clustered by PHYLIP package based on their ANI. Ncbi taxonomic assignation is represented in red, refseq status of the genomes in blue, the distance matrix represents the pairwise ANI of the genomes

Figure S2: Inhibitory diameters and MICs of ceftriaxone and cefotaxime using MIC gradient gradient strips of the 17390 *R. mucosa* strain.

Abbreviations: TX: ceftriaxone; CTX: cefotaxime

Ceftriaxone and cefotaxime have large diameters according to the disk diffusion method (36 mm and 27 mm, respectively). The MICs defined with the MIC gradient strip method were CRO = 3 mg/L and CTX > 32 mg/L.Figure S3: Distribution between DD and BMD for the *R. mucosa* cohort according to the CASFM-EUCAST 2024 breakpoints regarding eleven molecules A) Piperacillin‒tazobactam; B) Temocillin; C) Ceftazidime; D) Fosfomycin; E) Gentamicin; F) Meropenem; G) Ertapenem; H) Imipenem; I) Ciprofloxacin; J) Tigecycline; K) Cotrimoxazole

The number of strains observed for each inhibition zone diameter is presented, alongside the indication of susceptibility (in green) or susceptibility at high dosage (yellow) resistance (in red), based on the broth microdilution method. The diameters of inhibition were interpreted via the EUCAST-CASFM 2024 breakpoints, and the minimum inhibitory concentrations were interpreted according to the breakpoints proposed in the “nonrelated species PK/PD breakpoints” section of the reference EUCAST-CASFM 2024.

Figure S4: Inhibition diameters and MICs of Cefotaxime using DDM and MIC gradient strips alone or combined with clavulanic acid on the 2733 clinical strain
